# Prognostic Impact of *KRAS* and *SMARCA4* Mutations and Co-Mutations on Survival in Non-Small Cell Lung Cancer: Insights from the AACR GENIE BPC Dataset

**DOI:** 10.3390/biomedicines13092142

**Published:** 2025-09-02

**Authors:** Peter Manolakos, Yu-Bo Wang, Janice Withycombe, Luigi Boccuto, Diana Ivankovic

**Affiliations:** 1 Healthcare Genetics and Genomics PhD Program, Clemson University, Clemson, SC 29634, USA; 2 School of Mathematical and Statistical Sciences, Clemson University, Clemson, SC 29634, USA; yubow@clemson.edu; 3 Department of Healthcare Genetics and Genomics, Clemson University, Clemson, SC 29634, USA; jswithy@clemson.edu (J.W.); lboccut@clemson.edu (L.B.); divanko@clemson.edu (D.I.)

**Keywords:** *KRAS*, *SMARCA4*, mutation, co-mutation, *KRAS*/*SMARCA4*, NSCLC, survival, prognostic, biomarker, AACR GENIE

## Abstract

**Background/Objectives**: *KRAS* mutations are among the most prevalent oncogenic drivers in non-small cell lung cancer (NSCLC), with their impact on survival influenced by co-mutations. *SMARCA4* mutations are increasingly associated with poor prognosis and can be classified as class 1 or class 2 mutations. This study evaluates the prognostic implications of *KRAS* and *SMARCA4* mutations, including their co-mutations and their impact on NSCLC patients by utilizing real-world evidence. **Methods**: A retrospective analysis was conducted using the AACR GENIE Biopharma Collaborative (BPC) NSCLC 2.0 dataset. NSCLC patients with *KRAS* mutations, *SMARCA4* mutations, or *KRAS*/*SMARCA4* co-mutations were identified. Survival outcomes were assessed using univariate and multivariate Cox proportional hazards models, incorporating key clinical variables such as sex, race, smoking history, and stage. **Results**: Among 659 NSCLC patients with *KRAS* or *SMARCA4* mutations analyzed, *KRAS* mutations were the most prevalent (79%, *n* = 518). *SMARCA4* mutations were identified in 14% of cases (*n* = 95) across two classes. Six percent (*n* = 41) with class 1 mutations and 8% (*n* = 54) with class 2. Neither *SMARCA4* class was associated with worse survival outcomes compared to *KRAS*-mutated patients (*p* = 0.438 & 0.720). Patients harboring *KRAS*/*SMARCA4* class 1 co-mutations (3%, *n* = 18) had significantly worse overall survival compared to those with *KRAS* mutations alone (hazard ratio [HR] = 3.23, *p* < 0.001). In contrast, *KRAS*/*SMARCA4* class 2 co-mutations (4%, *n* = 28) did not significantly impact survival compared to *KRAS*-mutated patients (HR = 1.34, *p* = 0.205). **Conclusions**: *KRAS*/*SMARCA4* class 1 co-mutations are associated with significantly worse overall survival compared to *KRAS*-mutated NSCLC patients. Our multivariate analysis demonstrates the critical need to incorporate routine next-generation sequencing (NGS) testing in managing NSCLC patients at the time of metastatic diagnosis, with particular emphasis on identifying *SMARCA4* mutation class as a potential prognostic biomarker in those with *KRAS* co-mutations.

## 1. Introduction

Lung cancer is the most prominent cause of cancer mortality globally among both men and women, with approximately 1.8 million deaths in 2023 [1,2]. In the US, approximately 350 people die each day from lung cancer, causing more deaths in 2020 than prostate, breast, and pancreatic cancers combined [3]. Non-small cell lung cancer (NSCLC) accounts for 84% of lung cancer cases, and the five-year survival rate was only 25% across all stages from 2013 to 2019 [4,5]. With the adoption of genomic testing and advances in targeted therapy treatment for patients with NSCLC, there has been some improvement compared to the standard of care in overall survival (OS) rates and progression-free survival [6,7]. However, there needs to be a better understanding of how established NSCLC driver mutations, passenger mutations, and co-mutations impact overall survival outcomes.

In Western countries, mutations in the Kirsten rat sarcoma viral oncogene homolog (*KRAS*) gene are the most identified driver mutation in non-squamous NSCLC, presenting in approximately 30% of adenocarcinoma cases [8]. *KRAS* mutations are associated with poor overall survival rates among patients diagnosed with NSCLC and have been determined to be a weak but valid prognostic biomarker [9]. In relation to the current standard of care in oncology treatments, *KRAS* mutations in NSCLC have shown mixed results as predictive clinical biomarkers for survival outcomes with immune checkpoint inhibitor (ICI) treatments [10]. More common *KRAS* co-mutations may impact therapy as retrospective analyses and critical reviews have posited that NSCLC patients with *KRAS*-Serine/Threonine Kinase 11 (*STK11*) co-mutations are likely to exhibit primary resistance to ICI treatments [11]. Questions arise when understanding the prognostic impact of other *KRAS* co-mutations with non-driver mutations in patients with NSCLC.

SWI/SNF-related, matrix-associated, actin-dependent regulator of chromatin, subfamily A, member 4 (*SMARCA4*) mutations are found in approximately 8–12% of NSCLC patients [12,13,14]. *SMARCA4*-mutated patients tend to present with adenocarcinoma, a smoking history, and a low frequency of epidermal growth factor receptor (*EGFR*)/*SMARCA4* co-mutations [12,13,15]. In addition, a comprehensive critical review encompassing 21 studies concluded that overall survival outcomes were worse for NSCLC patients with *SMARCA4* mutations than those with wild-type *SMARCA4* alleles (tumors without *SMARCA4* mutations) [16].

*SMARCA4* mutations are categorized into two classes, as described by Schoenfeld et al., both of which are associated with epigenetic dysregulation but affect chromatin remodeling through distinct mechanisms [13,17]. For example, class 1 mutations involve truncating mutations (i.e., frameshift, nonsense), fusions, and homozygous deletions. *SMARCA4* encodes Brahma-Related Gene 1 (BRG1) protein, and class 1 mutations are often associated with BRG1 loss and loss of function [16,18]. Class 1 mutations are mainly observed in NSCLC, and some cancer cases indicate that this loss of function leads to reduced chromatin accessibility and diminished remodeling activity [19]. Class 2 mutations include missense mutations, which are suggested to induce gain-of-function or dominant-negative effects, possibly leading to deleterious cellular processes.

Two of the most extensive *SMARCA4* mutation datasets categorized these classes and types of mutations in a similar percentage of patients. For example, in the analysis conducted by Schoenfeld et al., including 408 *SMARCA4*-mutated NSCLC patients, 52% were class 1 and 48% were class 2 [13]. In this analysis, patients with *SMARCA4* class 1 mutations had a worse prognosis and OS than patients with class 2 mutations or who were *SMARCA4* wild-type (*p* < 0.001). Moreover, the landmark analysis conducted by Dagogo-Jack et al. of 3188 *SMARCA4*-mutated patients yielded similar distributions, with 49% of *SMARCA4*-mutated patients being categorized as class 1 and 51% of patients as class 2 [12].

In the American Association for Cancer Research (AACR) Project Genomics Evidence Neoplasia Information Exchange (GENIE) Biopharma Collaborative (BPC), *SMARCA4*-mutated NSCLC patients have an approximate 30% incidence of *KRAS*/*SMARCA4* co-mutations [20]. Moreover, *KRAS*/*SMARCA4* co-mutated patients across multiple NSCLC studies were found to have inferior survival outcomes throughout various types of cancer treatment analysis, including chemotherapy, ICI, and targeted therapy [21,22,23,24,25]. These inferior survival outcomes were also reinforced by the most extensive real-world evidence (RWE) retrospective NSCLC analysis of switch/sucrose non-fermenting (*SWI*/*SWF*) mutations, including *SMARCA4* variants. Investigators found that the co-mutated *KRAS*/*SMARCA4* NSCLC patients had worse survival vs. *KRAS-mutated*/*SMARCA4* wild-type patients (hazard ratio [HR] = 1.882, *p* < 0.00001) [14]. In contrast, one large analysis has demonstrated superior OS for NSCLC patients with *SMARCA4* mutations treated with ICI [13].

A limited number of *SMARCA4*-mutated NSCLC survival analyses have been completed, directly comparing class 1, class 2, and *SMARCA4* wild-type patients [13,19,21]. As previously noted, there are several analyses comparing *KRAS*/*SMARCA4* co-mutated to *KRAS*-mutated/*SMARCA4* wild-type patients [21,22,23,24,25]. To our knowledge, only one large multivariate analysis has comprehensively assessed *KRAS*/*SMARCA4* class 1 and *KRAS*/*SMARCA4* class 2 vs. *KRAS-mutated*/*SMARCA4* wild-type patients [13]. Analyzing high-quality observational RWE phenotypic data alongside genomic mutations and co-mutations in NSCLC may better elucidate how *KRAS*, *SMARCA4* (class 1 and 2), and *KRAS*/*SMARCA4* co-mutations intersect and influence overall patient outcomes and survival, thereby informing future drug development. This study aims to retrospectively analyze the prognostic impact on survival outcomes of *KRAS*-mutated and *SMARCA4* (class 1 and 2) mutated or *KRAS*/*SMARCA4* co-mutations in NSCLC patients via the AACR GENIE BPC dataset.

## 2. Materials and Methods

We utilized the AACR GENIE BPC NSCLC 2.0-public cohort, a publicly available dataset in cBioportal, to identify all NSCLC patients with *KRAS* mutations, *SMARCA4* mutations, or *KRAS*/*SMARCA4* co-mutations [26,27]. The NSCLC BPC cohort comprises 2004 samples from 1846 patients, randomly selected from samples in the GENIE 11.1-public release. The Dana–Farber Cancer Institute, Memorial Sloan Kettering Cancer Center, University Health Network, and Vanderbilt-Ingram Cancer Center contributed these samples. To be included in this analysis, patients were required to have at least 2 years of follow-up and a genomic sequencing report performed between 1 January 2014, and 31 December 2017. Clinical information was abstracted at each institution using the PRISSMM framework, deidentified, and provided to AACR for compilation [28]. The dataset of *KRAS* and *SMARCA4* mutations included all NSCLC histological subtypes and stages in the BPC cohort (i.e., lung adenocarcinoma and lung squamous cell carcinoma). The study was reviewed by Clemson University (Institutional Review Board #2023-0636) and deemed exempt as it utilized a publicly available database containing de-identified patient data.

*SMARCA4* class 1 and class 2 mutation categorizations were defined following the landmark analysis by Schoenfeld et al. [13]. *SMARCA4*-mutated patients with a frameshift deletion, frameshift insertion, nonsense mutation, fusion, splice site mutation, or splice region mutation were categorized as *SMARCA4* class 1 mutations. Patients with missense mutations, in-frame insertions, or deletions were classified as *SMARCA4* class 2 mutations. Tumors with concurrent *SMARCA4* class 1 and *SMARCA4* class 2 mutations were categorized as class 1. When multiple samples carrying the same mutation were attributed to the same patient ID, one sample was used for analysis, and the other samples were excluded. Similarly, patients with both *KRAS* and *SMARCA4* mutations were merged under a single de-identified patient ID to avoid duplication in the analysis.

Smoking history was classified into three groups: Never, Current, or Former user. Former users were combined [(quit < 1 year), (quit > 1 year), or (quit = unknown time)]. Disease stages were classified into Stage IV vs. Stage I–III. Any patients that were Stage I–III Not Otherwise Specified (NOS) were classified as Stage I–III. Patients were either categorized as White or Non-White. Non-White patients included Black, Chinese, Other Asian, American Indian, or Other. Patients with “unknown” defined variables for categories in scope were excluded from the analysis to maintain the accuracy and interpretability of the results. A very low number of samples fell into the unknown category: race (*n* = 18), stage (*n* = 1), and smoking history (*n* = 2).

### Statistical Methods

Statistical analyses were performed under the R software 4.4.1, and the statistical significance level was set at α = 0.05. To assess the marginal association of each risk factor with survival status (the event is deceased), the t-test and chi-square test were first conducted separately for a continuous (i.e., age and categorical (i.e., sex) factor. The results were presented along with other vital descriptive statistics (i.e., sample means and frequencies). Then, the Cox proportional hazards model was fitted to present the final findings jointly for all these factors (i.e., their joint associations with the deceased).

## 3. Results

After applying inclusion and exclusion criteria, 659 patients were retained in the dataset.

### 3.1. Clinical and Genomic Characteristics

Table 1 describes the clinical and genomic patient characteristics of the 659 patients with NSCLC. The mean age for next-generation sequencing (NGS) was 67.4 years. There were more females (*n* = 397, 60%) than males (*n* = 262, 40%). The most prevalent race was White (*n* = 600, 91%) vs. non-White/Other (*n* = 59, 9%), with Black (*n* = 32) and Chinese/Other Asian (*n* = 16) as the most common non-White patients. Forty-one percent of patients (*n* = 267) had Stage IV NSCLC vs. 59% of patients (*n* = 392) having early or locally advanced disease (Stage I-III). For early or locally advanced disease, Stage I was the most common (*n* = 192), followed by Stage II (*n* = 74), Stage III (*n* = 120), and Stage I–III NOS (*n* = 6). The vast majority of patients had a smoking history: former (*n* = 496, 75%) and current (*n* = 113, 17%) vs. never having a smoking history (*n* = 50, 8%).

Regarding the *KRAS* and *SMACAR4* mutation status for all 659 NSCLC patients, *KRAS* (*n* = 518, 79%) was the most common (Table 1). There were more *SMARCA4* class 2 mutations (*n* = 54, 8%) than *SMARCA4* class 1 mutations (*n* = 41, 6%). Similarly, slightly more *KRAS*/*SMARCA4* class 2 co-mutations (*n* = 28, 4%) than *KRAS*/*SMARCA4* class 1 co-mutations (*n* = 18, 3%) were reported. Seven *SMARCA4* class 1 patients had more than one type of *SMARCA4* mutation and were classified as class 1 due to one of the mutations being a frameshift or nonsense mutation. One *KRAS*/*SMARCA4* co-mutated patient had more than one type of *SMARCA4* mutation and was classified as *KRAS*/*SMARCA4* class 1 because they had a *SMARCA4* frameshift deletion and a *SMARCA4* missense mutation.

### 3.2. Univariate OS Analysis

Table 2 provides the clinical and genomic univariate analysis of OS status (living vs. deceased) from cancer diagnosis. Factors of interest were evaluated for their marginal associations with survival status. Significant associations with the risk of death were found under α = 0.05 in the factors “Stage” (*p* < 0.001) and “Mutation Type” (*p* = 0.001). While the mean ages of NGS sequencing were 66.7 and 68.0 years for living and deceased patients, respectively, the difference in the mean ages is insignificant (*p* = 0.085). Additionally, no significant OS differences were found between the sexes (female vs. male, *p* = 0.327) or racial groups (White vs. Non-white/Other, *p* = 0.713) or even among patients with varied smoking histories (Never vs. Former vs. Current smokers, *p* = 0.901).

### 3.3. Multivariate Analysis

Table 3 presents the results of the Cox regression model for patients with *KRAS*, *SMARCA4*, or *KRAS*/*SMARCA4*-co-mutated NSCLC. In this joint modeling, three factors were identified as important predictors accounting for a significantly increased risk of death. Specifically, “Stage IV” patients had an approximately four-fold risk of death than the earlier stages (HR = 4.01; 95% CI: 3.21–5.01; *p* < 0.001). *KRAS*/*SMARCA4* (class 1) co-mutated patients had a 3.2-fold risk of death compared to *KRAS* mutated patients (HR = 3.23; 95% CI: 1.90–5.51; *p* < 0.001). The age at sequencing was also associated with an increased risk of death (HR = 1.02; 95% CI: 1.01–1.03; *p* = 0.005).

Sex and race were not significantly associated with the risk of death [(Male vs. Female: HR = 1.18; 95% CI: 0.941–1.47; *p* = 0.153) and (White vs. Non-White/Other: HR = 0.978; 95% CI: 0.676–1.41; *p* = 0.905)]. Similarly, smoking history showed no significant association [(Former smoker vs. Never smoker: HR = 1.08; 95% CI: 0.702–1.66; *p =* 0.732) and (Current smoker vs. Never smoker: HR = 1.21; 95% CI: 0.745–1.98; *p* = 0.437)].

**Figure 1:**
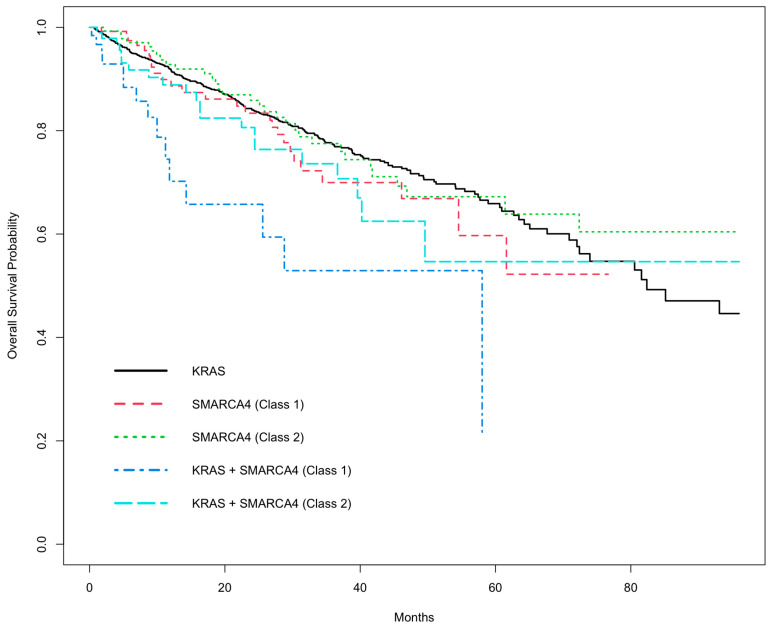
Adjusted Kaplan–Meier overall survival analysis among NSCLC patients with *KRAS*, *SMARCA4*, and *KRAS*/*SMARCA4* co-Mutations.

The other comparisons among the genomic mutation types were not associated with a significantly increased risk of death. Neither class 1 nor class 2 *SMARCA4* mutations had significantly worse OS compared to *KRAS* mutations: [*SMARCA4* (class 1) vs. *KRAS* (HR = 1.18; 95% CI: 0.779–1.78; *p* = 0.438)] and [*SMARCA4* (class 2) vs. *KRAS* (HR = 0.932; 95% CI: 0.635–1.37; *p* = 0.720)]. The comparison between patients with *KRAS*/*SMARCA4* (class 2) co-mutations vs. *KRAS* mutation was not significant (HR = 1.34; 95% CI: 0.851- 2.12; *p* = 0.205). To summarize these findings visually, the adjusted Kaplan–Meier curves (adjusted for other risk factors) for these five mutation types are shown in Figure 1.

## 4. Discussion

Our retrospective RWE analysis provides a comprehensive view of the prognostic impact of *KRAS* and *SMARCA4* mutations, including their co-mutations, on OS in patients with NSCLC. Utilizing the AACR GENIE BPC dataset, we identified distinct differences in genomic profiles influencing prognostic outcomes across a multivariate analysis. Notably, our findings highlight how these mutations and their co-mutations are significantly associated with increased patient risk, offering potential insights into how they may influence overall survival outcomes in NSCLC.

The results of the Cox-regression multivariate analysis demonstrated that *KRAS*/*SMARCA4* class 1 co-mutations are associated with worse OS compared to *KRAS* mutations for patients with NSCLC (HR = 3.23; 95% CI: 1.90–5.51; *p* < 0.001). In contrast, *KRAS*/*SMARCA4* class 2 co-mutations did not appear to confer a significantly worse OS than *KRAS* mutations (HR = 1.34; 95% CI: 0.851–2.12; *p* = 0.205). In addition, *SMARCA4* class 1 and class 2 mutations alone were not associated with worse OS when compared to *KRAS*-mutated NSCLC patients.

Our findings support the possible need to determine the *SMARCA4* mutation class as a prognostic factor when a patient carries a *KRAS*/*SMARCA4* co-mutation. Moreover, a recent meta-analysis outside of the co-mutation scope posited that *SMARCA4*-mutated class 1 was associated with worse OS for NSCLC patients (HR = 1.63; 95% CI: 1.44–1.85; *p* < 0.00001) in contrast to *SMARCA4*-mutated class 2 where no OS association was observed (HR = 1.34; 95% CI: 0.87–2.06; *p* = 0.18) [29]. This meta-analysis by Wankhede et al. differs from our OS analysis because it did not directly compare *KRAS*/*SMARCA4* co-mutated patients across both *SMARCA4* mutation classes.

Although multiple NSCLC studies demonstrate worse OS in patients with *KRAS*/*SMARCA4* co-mutations compared to *KRAS*-mutated patients, most do not analyze the impact of *SMARCA4* class 1 versus class 2 on OS within this co-mutated population [22,24,25]. For example, in the most extensive RWE analysis to date, Herzberg underscored the unfavorable prognostic effect of *KRAS*/*SMARCA4* co-mutations compared to *KRAS* mutations (HR = 1.882, *p* < 0.00001), and the *SMARCA4* mutation class effect was not directly noted [14]. They took a different approach and categorized *SWI*/*SWF* mutations as likely pathogenic or pathogenic (LP/P) per OncoKB, a precision oncology mutation database that provides the clinical significance of cancer-related mutations. Fifty-two percent of the *SMARCA4* mutations used for their analysis were deemed LP/P and most likely included some missense mutations, as OncoKB does categorize specific types of missense mutations as LP/P. In contrast, our analysis categorized all missense mutations as class 2 regardless of LP/P status following the methodology applied by Schoenfeld et al. [13].

Similar to our results, Schoenfeld et al. reported that NSCLC patients with *KRAS*/*SMARCA4* class 1 co-mutations were significantly associated with poorer survival (HR = 1.59; 95% CI: 1.04–2.41; *p* < 0.001) compared to *KRAS*-mutated/*SMARCA4* wild-type tumors [13]. Additionally, their NSCLC analysis showed that *KRAS*/*SMARCA4* class 2 co-mutations were associated with worse OS (HR = 2.75; 95% CI: 1.84–4.11; *p* < 0.001) compared to *KRAS*/*SMARCA4* wild-type tumors. These results remained prognostic after accounting for variables such as age, sex, histology, smoking status, TMB, and the presence of *STK11* or Kelch-like ECH-associated protein 1 *(KEAP1)* co-mutations.

### 4.1. Biological Insights

These survival findings reinforce the hypothesis that the loss-of-function nature of *SMARCA4* class 1 mutations may lead to diminished chromatin accessibility and remodeling, impairing gene regulation and potentially contributing to tumor progression [30]. As demonstrated in the Dagogo-Jack et al., Alessi et al., and Schoenfeld et al. studies, most of the class 1 *SMARCA4* truncating mutations result in a loss of BRG1 protein function [12,13,21]. For example, in these three studies, 84% (26 of 31), 100% (11 of 11), and 81% (50 of 62) NSCLC samples with truncating *SMARCA4* mutations lacked BRG1 immunohistochemistry (IHC) protein expression, respectively. These variants leading to BRG1 protein loss co-mutated with *KRAS* may lead to cancer treatment resistance by creating an immunosuppressive tumor microenvironment and impairing the effectiveness of DNA repair mechanisms. Liu et al. found that *KRAS*/*SMARCA4* co-mutated patients had significantly lower activated Cluster of Differentiation (CD4+) memory T cells (*p* = 0.0035) and (CD8+) T-cells proportions (*p* = 0.015) than *KRAS*-mutated/*SMARCA4* wild-type NSCLC patients [25].

### 4.2. Emerging Therapeutic Strategies for SMARCA4-Mutated NSCLC

Currently, there are several therapeutic approaches targeting *SMARCA4* mutations. SWI/SNF-related, matrix-associated, actin-dependent regulator of chromatin, subfamily A, member 2 (*SMARCA2*) selective protein degraders, such as PRT3789, offer a unique approach by exploiting synthetic lethality in *SMARCA4*-deficient tumors with >1000-fold selectivity for *SMARCA4*-mutated cancer cells compared to wild-type cells [31]. Two patients with NSCLC treated with PRT3789 had a confirmed partial response [32]. In treatment combination approaches, Ataxia–Telangiectasia and Rad3-related protein (ATR) inhibitors are being studied in combination with ICI treatment in previously treated NSCLC patients [33]. Tuvusertib (M1774) is a selective ATR inhibitor with antitumor activity as monotherapy in preclinical models with DNA damage repair pathway gene mutations. It is being studied with cemiplimab in a cohort of NSCLC patients with *SMARCA4* mutations [33,34].

### 4.3. Clinical Implications

Our multivariate analysis underscores the critical need to incorporate routine NGS testing in managing NSCLC patients at the time of metastatic diagnosis, with particular emphasis on determining *SMARCA4* mutation class in those with *KRAS* co-mutations. We demonstrated that NSCLC patients with *KRAS*/*SMARCA4* class 1 co-mutations were significantly associated with a worse prognosis and overall survival compared to *KRAS*-mutated patients. We also confirmed that patients diagnosed with Stage IV disease, across the entire cohort, had significantly worse prognosis and survival compared to those with Stage I–III disease.

### 4.4. Limitations

In addition to our study’s strengths, several limitations exist. This study was retrospective, hypothesis-generating, and subject to potential biases associated with real-world evidence. The multivariate analysis focuses on prognosis and overall survival risk and does not include specific treatment regimens or other potential *KRAS* co-mutations that could affect survival outcomes.

## 5. Conclusions

This analysis provides valuable insights into the prognostic impact of *KRAS* and *SMARCA4* mutations, including their co-mutations, on survival outcomes in metastatic NSCLC. Utilizing the AACR GENIE BPC NSCLC 2.0-public dataset, we demonstrated that *KRAS*/*SMARCA4* class 1 co-mutations were significantly associated with worse overall survival compared to *KRAS* mutations alone in NSCLC patients. These findings reinforce the importance of routine genomic profiling in NSCLC patients to optimize precision oncology treatment strategies further.

Our results highlight the biological complexity of *SMARCA4* class 1 versus class 2 mutations and their role in tumor progression when co-mutated with *KRAS* in patients with NSCLC. Future prospective studies should include *KRAS*/*SMARCA4* class 1 and *KRAS*/*SMARCA4* class 2 cohorts to validate the prognostic effects and potential predictive value of these co-mutations on survival outcomes in this high-risk NSCLC population.

## Figures and Tables

**Table 1:** Clinical and genomic characteristics of *KRAS*, *SMARCA4*, and *KRAS*/*SMARCA4* co-mutated NSCLC patients.

Clinical	N = 659, *n* (%)
	
**Age at NGS sequencing (mean years)**	67.4
**Sex**	
Female	397 (60%)
Male	262 (40%)
	
**Race**	
Non-White/Other	59 (9%)
White	600 (91%)
	
**Stage**	
I–III (early and locally advanced)	392 (59%)
IV (metastatic)	267 (41%)
	
**Smoking History**	
Never	50 (8%)
Former	496 (75%)
Current	113 (17%)
	
**Mutation Type**	
*KRAS*	518 (79%)
*SMARCA4* (class 1)	41 (6%)
*SMARCA4* (class 2)	54 (8%)
*KRAS + SMARCA4* (class 1)	18 (3%)
*KRAS + SMARCA4* (class 2)	28 (4%)

**Abbreviations**: *KRAS*, Kirsten rat sarcoma viral oncogene homolog; NGS, next-generation sequencing; NSCLC, non-small cell lung cancer; *SMARCA4*, SWI/SNF-related, matrix-associated, actin-dependent regulator of chromatin, subfamily A, member 4.

**Table 2:** Univariate analysis of OS status from cancer diagnosis for *KRAS*, *SMARCA4*, and *KRAS*/*SMARCA4* co-mutated NSCLC patients.

OS Status from Cancer Diagnosis	Total	Living	Deceased	*p*-Value
				
**Clinical**	N = 659	N = 311	N = 348	
				
**Age at NGS sequencing (mean)**		66.7	68.0	0.085
				
**Sex**				0.327
Female	397	194	203	
Male	262	117	145	
				
**Race**				0.713
Non-White/Other	59	26	33	
White	600	285	315	
				
**Stage**				**<0.001**
I–III (early and locally advanced)	392	253	139	
IV (metastatic)	267	58	209	
				
**Smoking History**				0.901
Never	50	25	25	
Former	496	232	264	
Current	113	54	59	
				
**Genomic**	N = 659			
				
**Mutation Type**				**0.001**
*KRAS*	518	264	254	
*SMARCA4* (class 1)	41	15	26	
*SMARCA4* (class 2)	54	22	32	
*KRAS + SMARCA4* (class 1)	18	3	15	
*KRAS + SMARCA4* (class 2)	28	7	21	

**Abbreviations:** *KRAS*, Kirsten rat sarcoma viral oncogene homolog; NGS, next-generation sequencing; NSCLC, non-small cell lung cancer; OS, overall survival; *SMARCA4*, SWI/SNF-related, matrix-associated, actin-dependent regulator of chromatin, subfamily A, member 4.

**Table 3:** Multivariate analysis of overall survival in NSCLC patients with *KRAS*, *SMARCA4*, and *KRAS*/*SMARCA4* co-mutations.

Factor	HR	95% CI	*p*-Value
**Sex**			
(Reference group: Female)			
Male	1.18	0.94–1.47	0.153
**Age**	1.02	1.01–1.03	**0.005**
**Race**			
(Reference group: Non-White/Other)			
White	0.98	0.68–1.41	0.905
**Smoking History**			
(Reference group: Never smoker)			
Former smoker	1.08	0.70–1.66	0.732
Current smoker	1.21	0.75–1.98	0.437
**Stage**			
(Reference group: Stage I–III)			
Stage IV	4.01	3.21–5.01	**<0.001**
**Genomic**			
(Reference group: *KRAS*)			
*SMARCA4* (class 1)	1.18	0.78–1.78	0.438
*SMARCA4* (class 2)	0.93	0.64–1.37	0.720
*KRAS + SMARCA4* (class 1)	3.23	1.90–5.51	**<0.001**
*KRAS + SMARCA4* (class 2)	1.34	0.85–2.12	0.205

**Abbreviations:** CI, Confidence Interval; HR, Hazard Ratio; *KRAS*, Kirsten rat sarcoma viral oncogene homolog; NSCLC, non-small cell lung cancer; OS, overall survival; *SMARCA4*, SWI/SNF-related, matrix-associated, actin-dependent regulator of chromatin, subfamily A, member 4.

## Data Availability

All data utilized in this analysis were obtained from the AACR Project GENIE BPC database. The dataset is publicly available through the AACR GENIE Data Commons (https://genie.cbioportal.org/). The specific dataset version employed is [Version 2.0-public]. Researchers can access the dataset following the instructions provided on the AACR GENIE website.
